# Beef Tea Versus True Food

**Published:** 1884-04

**Authors:** 


### BEEF TEA VERSUS TRUE FOOD.

THE PROGRESS of Science is marked by the debris of the wrecks of many erroneous beliefs and superstitions, and in no direction is this fact more marked than in the application of science to the affairs of every-day life. Among those demolished beliefs is that which attached to beef tea a high degree of nutritive power. We do not know, nor do we care to inquire at this time, whether it had its origin with the people and was endorsed by the profession, or whether it originated with the latter, who communicated it, with much else which science has demolished, to the confiding public. Suffice it to say that within a comparatively few years (too few to have, as yet effected its complete overturning) the belief that beef tea was a form of concentrated nutriment, was very general among both the public and physicians.

Doubtless, under this delusion, multitudes of sick sufferers, tenderly cared for by those to whom they were dearer than all else, have been literally starved—starved to death with their stomachs full of beef tea.

Philosophy has demonstrated that *the* nutritious principle of all food is either albumen, as in animal food, or albuminoid matter (gluten), as in vegetable diet While starch and fat and the inorganic salts each play their part in nutrition, the nutriment, animal or vegetable, which shall sustain life by repairing waste, must contain albuminoid matter ; the degree of its nutritive value being in a direct ratio to this constituent. Applying this incontestable fact to the value of beef tea, we find this substance practically destitute of power to nourish. It is, as might readily be surmised before-hand, from a knowledge of the method of its preparation, wanting in albumen. Albumen coagulates at a temperature of 160° F., and when coagulated, is insoluble in water. As it exists in beef, it is very intimately related to the fibre; is, indeed, a constituent of it. It will be readily understood, therefore, that when it is coajulated in this relation, it must be removed with the beef which has been employed in the preparation of beef tea.

Now, beef tea, as it is prepared, has been subjected in the process, to a heat much in excess of 155° F. Ordinarily, boiling water (212° F.) is poured directly on the beef ; there is a plan of adding cold water, and afterwards slowing, raising its temperature, or "digesting," as it is sometimes improperly termed. Beef tea, regardless of the manner of its preparation, will be found, on the application of proper chemical tests, to be destitute, or nearly so, of albumen ; that essential nutritive principle having been cast aside with the beef, from which the "strength" is supposed to have been extracted. It contains at best only the inorganic salts of the beef, which are slightly stimulating in their action, but are not nutrients.

What is required in the sick room is a preparation of beef from which a true liquid food can be prepared. Such a preparation must have been made without sufficient heat to coagulate albumen, which substance must have been rendered soluble by previous digestion in hot water. Such a preparation added to hot water, makes an agreeable, highly-nutritious and easily-assimilated food. It was long a desideratum in medicine, and its discovery has severely taxed the ingenuity of the chemist, since the exposure of the beef tea fallacy demonstrated the necessity of it. It has at length been supplied in the preparation known as "Sarcopeptones," a preparation for the discovery of which the medical profession and the public have been placed under obligation to Dr. Rueisch. The addition of the "Sarcopeptones" to hot water gives a solution highly albuminous, palatable and nutritious. The name is explanatory of the nature of the preparation, being compounded from two Greek words signifying digested flesh.

The beef is "peptonized," which is a part of the process of digestion. The peptonization having been done outside the stomach, that organ, weakened, as it is prone to be, in disease, is relieved of what would be to it, under the circumstances, a severe burden. "Sarcopeptones" is a most practical illustration of the inestimable service which science may render in the practical affairs of life. But there are cases in the experience of all physicians, where the condition of patients absolutely demands nourishment, and where the stomach is too weak to tolerate even the blandest foods. In these cases we have, as an eminent London physician expresses it, "an effective second stomach in the rectum;" and moreover, the inconvenient method of introducing foods and medicines into the rectum by syringes, has been superceded by the invention of *Hollow Suppositories* of various capacities, from three drops to half an ounce.

The new form of these suppositories is easily applied, and the pure butter of cacao, from which they are made, is, of itself, an excellent nutriment.

---

* Manufactured by Parker, Davis & Co., Detroit. To be obtained of any druggist.

